# Trends and patterns of life satisfaction and its relationship with social support in Canada, 2009 to 2018

**DOI:** 10.1038/s41598-022-13794-x

**Published:** 2022-06-12

**Authors:** Yingying Su, Carl D’Arcy, Muzi Li, Xiangfei Meng

**Affiliations:** 1 Department of Psychiatry, Faculty of Medicine and Health Sciences, McGill University, 6875 Boulevard LaSalle, Montreal, QC H4H 1R3 Canada; 2 Douglas Research Centre, Montreal, QC Canada; 3 School of Public Health, University of Saskatchewan, Saskatoon, SK Canada; 4 Department of Psychiatry, College of Medicine, University of Saskatchewan, Saskatoon, SK Canada

**Keywords:** Public health, Quality of life

## Abstract

The present study aims to explore the trends and patterns of life satisfaction in Canada from 2009 to 2018 and to examine changes in the associations between social support and life satisfaction over time. Data were from ten annual Canadian Community Health Surveys (CCHS). Each survey represents 97% of the Canadian population. Point estimates and 95% confidence intervals (CIs) of life satisfaction were calculated at the population level. Generalized linear regression was used to explore the relationship between life satisfaction and social support both nationally and in different population subgroups. The annual life satisfaction score gradually increased both at national and provincial levels from 2009 to 2018. Individuals who were women, aged between 12 and 19 years, living in rural areas, were most satisfied with their lives. There was a positive correlation between social support and life satisfaction for the provinces and the study years for which information on social support was available. Our findings suggest strengthening social support could be a public health target for promoting greater life satisfaction. Timely availability and analysis of life satisfaction and social support data could better inform policy and promote wellbeing at a population level.

## Introduction

Life satisfaction measures how people evaluate their life as a whole^1^. It is frequently used as a determinant of health and well-being^2^, which includes a wide range of adaptive life outcomes, such as health, longevity, quality interpersonal relationships, and vocational success^3^. A society’s level of subjective well-being is intimately related to the legitimacy of the socioeconomic and political system and could significantly influence the stabilization of the societal and political order of the society^4^.

Life satisfaction has been shown to vary between nations, with wealthy nations reporting higher rates of life satisfaction as opposed to poorer nations indicating that living conditions strongly influence life satisfaction^5,6^. The Organization for Economic Co-operation and Development (OECD) has adopted the measure of life satisfaction to compare well-being and changes in well-being between nations over the past 20 years^7^. That research shows life satisfaction is not evenly shared across the OECD countries, but the average life satisfaction score in OECD countries slightly increased in 2018 compared to the score in 2010^8^. Canada had a relatively high level of life satisfaction compared to other OECD countries^9^. Its average life satisfaction score varied from 7.6 to 8.3 between 2003 and 2011^10^. A later study conducted by Lu et al.^11^ reported that the average life satisfaction score in Canada ranged from 7.8 to 8.2 between 2009 and 2013.

Although life satisfaction has been recognized as an important topic in happiness studies and has attracted attention from a wide range of disciplines, particularly psychology, economics, sociology, and political science^12^, there have not been many studies conducted to examine the trends of life satisfaction at a national level in the context of the significant economic and societal changes that have occurred in recent years. It is vital to have updated information on the trends and patterns of life satisfaction at a national level^13^. When people feel their lives have improved it helps engender trust in socio-economic and political institutions, whereas if life satisfaction decreases over time, it may destabilize the sociopolitical order^4^.

Identification of determinants and correlates of life satisfaction can provide important insights into targets that can be potentially used to improve life satisfaction at a national and local level^14^. Some population characteristics associated with high levels of life satisfaction such as social capital, as measured by the strength of family, neighborhood, religious and community ties have been consistently studied in international comparisons^15^. Social support is an important positive psychological attribute for positive mental health and a component of social capital that has not been extensively studied in the life satisfaction literature^16^. It refers to the provision or the exchange of emotional, instrumental, or informational resources by non-professionals, in the context of a response to the perception that others need it^17^. Social support could be further characterized by affective support (i.e., love, respect), confirmation (i.e., confirming the moral and factual “rightness” of actions and statements), and practical help (i.e., aid in work, giving information). These various aspects of social support are usually highly interrelated^17,18^. The literature has accumulated ample evidence to support the association between social support and life satisfaction, but most of the evidence can only provide a snapshot of this relationship at a given time point and a societal level^19–21^. What has been missing is an updated analysis of trends in life satisfaction over time at a national level and for different population sub-groups. There is also little known about how social support, and changes in social support, are linked with changes in life satisfaction over time and in different social contexts.

Large-scale longitudinal studies, as well as repeated cross-sectional studies, are needed to articulate how and to what extent social support relates to life satisfaction over time. We were not aware of any study that had reported the association between social support and life satisfaction using large national datasets. Considering the implementation difficulty and operability of large-scale longitudinal studies, repeated cross-sectional studies have been relatively easier to be carried out and could provide excellent data about how social support and life satisfaction have changed over time. Such findings could inform public health campaigns focusing on specific subgroups of the general population and/or specific modifiable risk factors to promote greater life satisfaction.

To fulfill these knowledge gaps in the literature, the present study aims to examine: 1) the trend and pattern of life satisfaction from 2009 to 2018 in Canada at the national and provincial levels and among key sociodemographic groups, and 2) test the relationships between social support and changes in life satisfaction among these subgroups over time. We hypothesized that the level of life satisfaction would increase over ten years and vary across different sociodemographic groups. Additionally, the level of social support would be positively correlated with life satisfaction during the study period.

## Methods

### Participants and procedure

**Figure 1:**
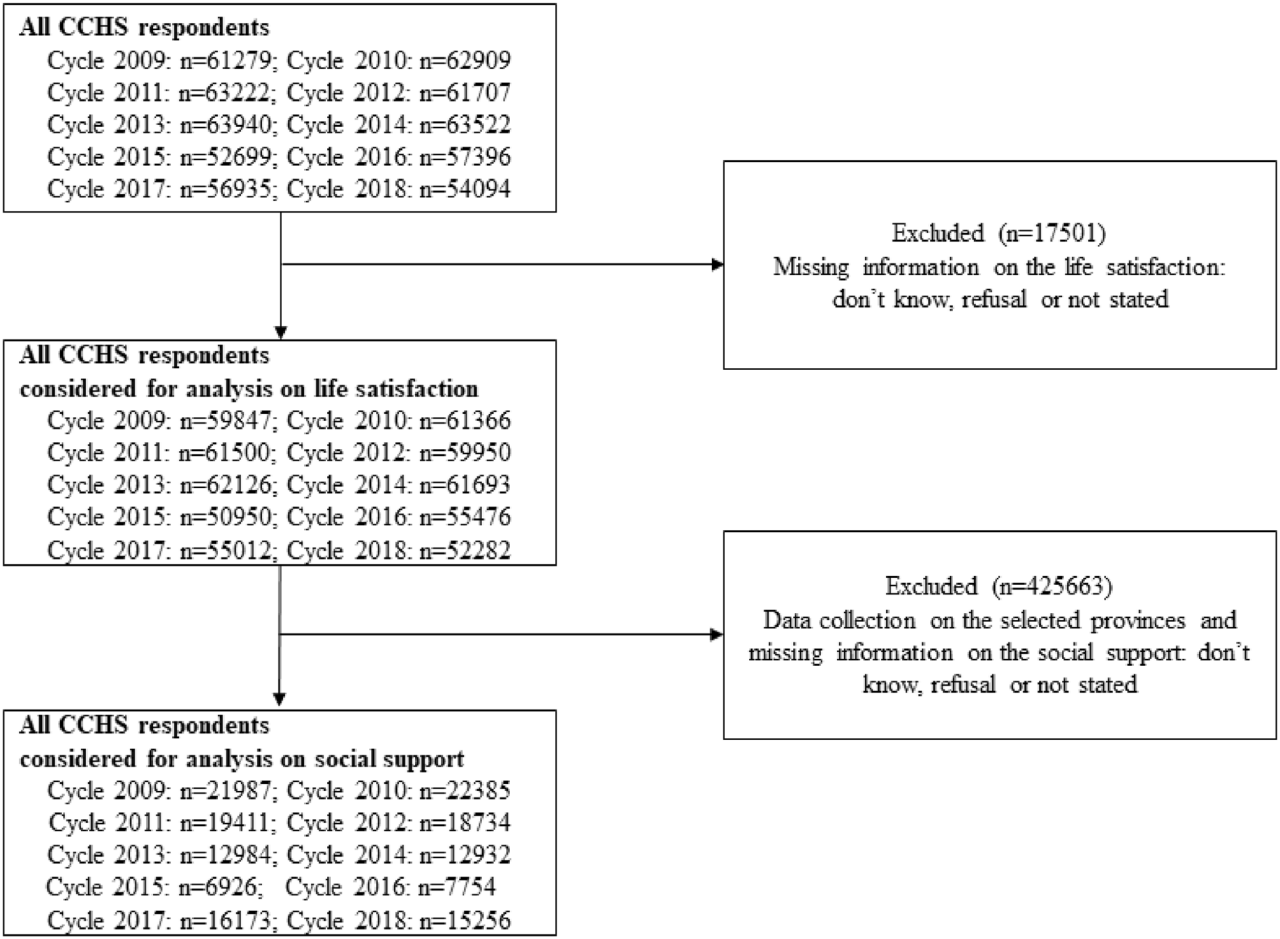
A flow chart of sample sizes included in the analyses.

Data analyzed are drawn from ten annual Canadian Community Health Surveys (CCHS) conducted by Statistics Canada covering the years from 2009 to 2018. The flow chart of the study sample is in Fig. 1. The CCHS surveys study representative samples of respondents aged 12 and over residing in the ten provinces and the three territories of Canada. The CCHS surveys use multistage cluster sampling of individuals within household clusters by health region strata. Data were collected using computer-assisted in-person and telephone interviewing. Participants in the original surveys signed informed consent and voluntarily participated in the survey. The surveys received ethical approval through Statistics Canada procedures. The present study was approved by the Faculty of Medicine Institutional Ethical Board, McGill University (#20-08-040). All research was performed in accordance with the Declaration of Helsinki.

### Instruments

*Life satisfaction* was measured by a single item: “How do you feel about your life as a whole right now?” using a scale of 0 to 10, where 0 means ‘Very dissatisfied’ and 10 means ‘Very satisfied’. This single-term scale has been widely used and seen as a reliable and valid indicator for individuals’ subjective well-being^22^. There are no standardized cut-off points for classifying or differentiating satisfied from dissatisfied life. A score of 9 or 10 has been considered to denote a high level of positive life satisfaction, and a score of 6 or less denoting a low level of life satisfaction^11^.

*Social support* was measured by the Social Support Availability (SSA) instrument between 2009 and 2010 and by the Social Provisions Scale-10 item (SPS-10) from 2011 onwards^23^. SSA included a total of 20 items concerning: tangible support, positive social interaction, emotional or informational support, and affection, which has been shown to have adequate levels of construct validity and reliability^24^. As suggested by prior research, a score of 76 was used to categorize social support as high or low^25^. SPS-10 has five subscales: attachment, guidance, reliable alliance, social integration, and reassurance of worth^26^. A score of 30 or above is considered as high social support^27^. The SPS-10 has been found to be a valid measure of social support with strong internal consistency and adequate test–retest reliability^28,29^. The Cronbach’s alpha for SSA was both 0.96 for the year of 2009 and 2010. The Cronbach’s alpha for SPS-10 ranged from 0.91 to 0.93 between 2011 and 2018.

### Covariates

Factors including *age, gender, province of residence, type of residential area, population centre size, marital status, educational attainment, total household income, immigrant status, self-perceived health, self-rated stress, physical activity, chronic conditions, type of smokers,* and *type of drinker*, were included in the analyses as covariates.

*Age* was categorized into seven groups, including 12–19 years, 20–29 years, 30–39 years, 40–49 years, 50–59 years, 60–69 years, and 70 years and more. *Marital status* was categorized as married/common-law, widowed/separated/divorced, and single. *Chronic health condition* was treated as a binary variable indicating whether the respondent had been diagnosed with at least one chronic health condition from a list including asthma, high blood pressure, diabetes, heart disease, cancer, stroke, chronic bronchitis, emphysema, and chronic obstructive pulmonary disease. *Smoking status* was categorized as: current smoker, former smoker, or non-smoker. *Type of drinker* was grouped as: non-drinker, moderate drinker, or binge drinker.

### Statistical analysis

Point estimates and 95% confidence intervals (CIs) of life satisfaction were calculated for the studied period (2009–2018). The bootstrap program and the sampling weights provided by Statistics Canada were used to account for the complex sampling design. The point estimates were used to illustrate the annual level of life satisfaction by its categories and its continuous score. We also stratified life satisfaction by gender, age, province, type of residential areas, and population size to explore its distribution. The age/gender-standardized point estimates of life satisfaction with their 95% CIs were also calculated for each socio-demographic subgroup based on the 2018 Canadian population. Generalized linear regression was used to explore the relationship between life satisfaction and social support after adjusting for a set of covariates in the national population and different population subgroups. All the analyses were performed using STATA software, version 9.0^30^.

### Ethics declarations

Participants in the original survey signed informed consent and voluntarily participated in the survey. The original survey received ethical approval through Statistics Canada procedures. The present study was approved by the Faculty of Medicine Institutional Ethical Board, McGill University (#20-08-040).

### Consent to participate

Informed consent was obtained from all individual participants included in the study.

## Results

### Trends and patterns of life satisfaction at a population level

Overall, the annual average score of life satisfaction among the Canadian population increased across the study period from 2009 to 2018. It ranged from 8.01 (on a scale with a maximum value of 10) in 2009 to 8.12 in 2018 with a peak of 8.14 in 2015 at the national level (Table 1). Women, aged between 12 and 19 years, living in rural regions, and those who resided in Newfoundland & Labrador and Prince Edward Island reported a higher level of life satisfaction (a score of 9 or 10) (Fig. S1–S4). There was a 3.2% increase in the proportion of people with a higher level of life satisfaction from 2009 (38.4%) to 2018 (41.6%) (Table 2). Respondents with a lower level of life satisfaction (score 6 or less) decreased from 12.4% in 2009 to 11.3% in 2018 (Table S1). Women consistently reported a higher level of life satisfaction across the study period. The proportion of a higher level of life satisfaction in men and women ranged from 37.0% to 41.3% and 39.8% to 42.0%, respectively. Overall, residents aged between 12 and 19 years were the largest group with a higher level of life satisfaction and the smallest proportion with a lower level of life satisfaction. We observed a ‘u-shape’ relationship between age and annual life satisfaction scores with middle-aged respondents having a lower level of life satisfaction whereas younger and older age groups reported higher levels of life satisfaction. Provincial differences in annual life satisfaction scores were also noted. During the study period, Newfoundland and Labrador had the largest proportion of residents with a higher level of life satisfaction, whereas British Columbia had the smallest proportion (45.5% vs. 37.6%). Consistently, Newfoundland and Labrador had the smallest proportion of residents with a lower level of life satisfaction whereas the Territories had the largest proportion of residents with a lower level of life satisfaction (10.2% vs. 15.0%). In terms of population centre size, rural areas had a bigger proportion of residents with a higher level of life satisfaction than small CMAs (Census Metropolitan Areas), which in turn had a larger proportion of residents with a higher level of life satisfaction than residents of large urban cores. The urban–rural gap in the proportion reporting high levels of life satisfaction gradually narrowed throughout the study period (6.6% in 2009 vs. 5.4% in 2018).

**Table 1:** Average life satisfaction score by population subgrouping, Canada, 2009–2018.

Variable	2009	2010	2011	2012	2013	2014	2015	2016	2017	2018
National level	8.01	8.01	7.99	8.01	7.99	8.00	8.14	8.11	8.11	8.12
**Gender**										
Men	7.98	7.98	7.98	7.99	7.96	7.97	8.12	8.08	8.08	8.13
Women	8.05	8.04	8.01	8.03	8.02	8.02	8.15	8.13	8.13	8.12
**Age (years)**										
12–19	8.31	8.29	8.31	8.32	8.34	8.32	8.58	8.52	8.59	8.52
20–29	8.10	8.13	8.03	8.02	8.04	8.01	8.17	8.11	8.10	8.11
30–39	8.05	8.06	8.04	8.09	8.02	7.99	8.15	8.17	8.14	8.14
40–49	7.87	7.87	7.88	7.87	7.81	7.90	8.02	8.03	8.04	7.97
50–59	7.89	7.86	7.84	7.93	7.84	7.91	8.00	7.99	7.94	7.99
60–69	8.03	8.01	7.98	8.01	8.02	8.02	8.12	8.04	8.02	8.15
70 +	7.94	7.92	7.98	7.91	8.01	7.94	8.06	7.99	8.10	8.12
**Province of residence**										
AB	7.96	7.98	7.98	7.99	8.00	8.05	8.18	8.10	8.08	8.04
BC	7.93	7.92	7.96	7.86	7.94	7.96	8.06	8.05	8.00	8.04
MB	8.01	7.98	7.98	8.00	8.03	8.07	8.13	8.02	8.18	8.07
NB	8.19	8.14	8.13	8.17	8.14	8.10	8.07	8.20	8.21	8.21
NL	8.16	8.25	8.31	8.12	8.35	8.19	8.27		8.19	8.24
NS	8.06	8.14	8.15	8.07	8.06	8.02	8.13	8.06	8.03	8.08
ON	7.95	7.96	7.92	7.99	7.93	7.96	8.12	8.06	8.09	8.12
PEI	8.29	8.27	8.11	8.18	8.13	8.19	8.30	8.25	8.19	8.17
QC	8.15	8.10	8.09	8.10	8.05	7.99	8.17	8.18	8.18	8.22
SK	8.10	8.13	8.09	8.06	8.10	8.23	8.25	8.26	8.20	8.16
Territories	8.00	8.05	7.97	7.96	7.98	7.92	8.03	8.04	–	–
**Residency area**										
Rural	8.17	8.19	8.17	8.20	8.16	8.15	8.33	8.30	8.29	8.26
Urban	7.98	7.97	7.96	7.97	7.95	7.96	8.10	8.07	8.07	8.09
**Population centre size**										
Rural area	8.18	8.17	8.16	8.22	8.14	8.15	8.33	8.30	8.27	8.25
Urban core	7.96	7.96	7.93	7.94	7.94	7.94	8.08	8.05	8.04	8.07
Urban fringe	8.13	8.04	8.08	8.08	8.12	8.14	8.06	8.31	8.26	8.24
Urban O/S CMA	8.13	8.06	8.08	8.14	8.09	8.16	8.24	8.13	8.18	8.20
Secondary urban core	8.05	8.07	8.01	8.08	7.94	8.21	8.23	8.11	8.26	8.22
Mix of Urban/rural	8.11	8.17	8.15	8.14	8.17	8.07	8.32	8.29	8.33	8.28

AB: Alberta; BC: British Columbia; MB: Manitoba; NB: New Brunswick; NL: Newfoundland and Labrador; NS: Nova Scotia; ON: Ontario; PEI: Prince Edward Island; QC: Quebec; SK: Saskatchewan; Urban O/S CMA: Urban center outside census metropolitan area.

**Table 2:** Annual life satisfaction with the score of nine and ten, Canada, 2009–2018.

Variable	2009	2010	2011	2012	2013	2014	2015	2016	2017	2018
	P% (95%CI)	P% (95%CI)	P% (95%CI)	P% (95%CI)	P% (95%CI)	P% (95%CI)	P% (95%CI)	P% (95%CI)	P% (95%CI)	P% (95%CI)
National level	38.4 (37.7, 39.1)	38.1 (37.4, 38.8)	37.4 (36.7, 38.2)	37.7 (37.0, 38.5)	37.7 (36.9, 38.4)	37.7 (37.0, 38.5)	42.2 (41.5, 43.)	41.7 (41.0, 42.4)	41.6 (40.9, 42.4)	41.6 (40.1, 42.4)
**Gender**										
Men	37.0 (36.0, 38.0)	37.0 (35.9, 38.0)	36.9 (35.8, 38.0)	36.7 (35.6, 37.8)	36.2 (35.1, 37.2)	36.3 (35.2, 37.4)	41.5 (40.4, 42.6)	40.5 (39.4, 41.5)	41.3 (40.3, 42.3)	41.3 (40.2, 42.4)
Women	39.8 (38.9, 40.7)	39.2 (38.2, 40.2)	38.0 (37.0, 38.9)	38.7 (37.7, 39.7)	39.1 (38.1, 40.1)	39.1 (38.1, 40.1)	43.0 (42.0, 44.0)	42.8 (41.8, 43.8)	42.0 (41.0, 43.0)	42.0 (40.9, 43.0)
**Age (years)**										
12–19	46.3 (44.5, 48.1)	44.1 (42.3, 45.9)	45.7 (43.8, 47.6)	45.5 (43.4, 47.5)	46.3 (44.3, 48.2)	45.0 (43.0, 47.0)	55.6 (53.4, 57.8)	54.2 (52.1, 56.4)	55.3 (53.1, 57.6)	54.5 (52.1, 56.8)
20–29	38.7 (36.9, 40.6)	38.9 (37.2, 40.7)	35.2 (33.4, 37.0)	36.1 (34.1, 38.0)	35.4 (33.6, 37.4)	34.6 (32.7, 36.6)	40.4 (38.2, 42.6)	39.0 (37.0, 41.0)	38.8 (36.8, 40.9)	38.5 (36.3, 40.8)
30–39	38.0 (36.4, 39.7)	38.5 (36.8, 40.3)	37.3 (35.5, 39.2)	38.5 (36.6, 40.5)	36.9 (35.0, 38.8)	36.3 (34.3, 38.3)	40.1 (38.2, 41.9)	41.3 (39.5, 43.1)	41.0 (39.3, 42.8)	41.1 (39.2, 43.1)
40–49	33.8 (32.0, 35.7)	34.1 (32.1, 36.1)	34.3 (32.3, 36.4)	33.8 (31.0, 35.9)	33.1 (30.9, 35.3)	35.1 (33.0, 37.3)	38.1 (36.1, 40.0)	38.9 (36.9, 41.0)	39.9 (38.0, 41.7)	36.6 (34.5, 38.7)
50–59	35.8 (34.1, 37.5)	36.1 (34.1, 38.2)	34.9 (33.0, 36.7)	35.8 (33.8, 37.8)	35.5 (33.7, 37.4)	36.7 (34.6, 38.7)	39.9 (38.0, 41.8)	39.3 (37.6, 41.1)	37.9 (36.1, 39.7)	38.7 (36.7, 40.7)
60–69	40.9 (39.4, 42.5)	39.8 (38.1, 41.5)	39.3 (37.6, 40.9)	39.1 (37.3, 40.9)	40.0 (38.5, 41.6)	39.9 (38.4, 41.5)	43.9 (42.2, 45.7)	42.0 (40.3, 43.7)	40.8 (39.2, 42.3)	43.9 (42.1, 45.6)
70 +	39.0 (37.5, 40.6)	38.1 (36.6, 39.6)	39.7 (38.0, 41.4)	38.9 (37.4, 40.4)	41.4 (39.8, 42.9)	40.1 (38.6, 41.6)	43.5 (41.8, 45.2)	41.5 (39.9, 43.1)	43.5 (42.0, 45.1)	43.5 (41.9, 45.1)
**Province of residence**										
AB	36.5 (34.4, 38.6)	37.9 (35.7, 40.1)	38.0 (35.8, 40.3)	36.8 (34.5, 39.2)	37.9 (35.5, 40.3)	38.7 (36.3, 41.1)	43.8 (41.8, 45.8)	40.5 (38.7, 42.4)	41.3 (39.6, 43.1)	38.4 (36.5, 40.4)
BC	37.4 (35.6, 39.3)	36.4 (34.5, 38.4)	36.5 (34.6, 38.4)	34.2 (32.2, 36.2)	36.2 (34.2, 38.2)	36.6 (34.7, 38.6)	40.3 (38.4, 42.1)	40.2 (38.4, 42.1)	37.9 (36.2, 39.7)	40.4 (38.5, 42.3)
MB	38.4 (35.4, 41.5)	38.3 (35.0, 41.8)	37.0 (33.9, 40.2)	40.5 (37.2, 43.9)	38.5 (35.6, 41.5)	39.1 (36.1, 42.2)	42.3 (39.5, 45.3)	40.2 (36.9, 42.5)	43.1 (40.2, 46.1)	39.4 (36.5, 42.3)
NB	45.6 (42.8, 48.6)	41.6 (38.8, 44.5)	40.3 (37.3, 43.5)	42.1 (39.0, 45.2)	43.5 (40.6, 46.4)	43.2 (40.3, 46.2)	42.5 (39.0, 46.1)	45.2 (41.9, 48.5)	44.8 (41.5, 48.1)	46.6 (43.4, 49.8)
NL	42.7 (39.6, 46.0)	45.9 (42.5, 49.3)	46.0 (42.6, 49.4)	41.2 (37.6, 45.0)	49.1 (45.7, 52.5)	42.1 (38.8, 45.4)	48.6 (44.9, 52.3)	49.9 (46.3, 53.5)	45.6 (42.1, 49.1)	44.2 (40.6, 47.8)
NS	42.9 (39.9, 46.0)	44.6 (41.4, 47.8)	41.4 (38.3, 44.6)	38.5 (35.3, 41.7)	39.1 (36.2, 42.0)	38.3 (35.5, 41.2)	42.8 (38.8, 45.8)	43.9 (41.1, 46.7)	42.5 (39.6, 45.4)	42.6 (39.8, 45.5)
ON	37.2 (36.1, 38.3)	37.0 (35.8, 38.2)	36.4 (35.1, 37.6)	37.5 (36.2, 38.8)	37.1 (35.9, 38.4)	37.8 (36.5, 39.1)	41.7 (40.3, 43.2)	41.4 (40.1, 42.7)	41.7 (40.3, 43.0)	41.7 (40.3, 43.2)
PEI	48.1 (43.7, 52.6)	45.1 (40.5, 49.8)	41.4 (36.8, 46.2)	41.6 (37.1, 46.3)	41.0 (36.6, 45.4)	41.1 (36.7, 45.6)	46.9 (42.6, 51.3)	44.9 (40.6, 49.3)	44.4 (40.2, 48.6)	42.0 (37.8, 46.3)
QC	39.9 (38.4, 41.5)	38.7 (37.2, 40.3)	38.0 (36.5, 39.5)	39.1 (37.5, 40.7)	37.5 (36.0, 39.0)	36.1 (34.6, 37.7)	42.3 (40.8, 43.7)	42.4 (40.9, 43.8)	42.5 (41.2, 43.9)	43.4 (42.0, 44.9)
SK	39.8 (37.3, 42.4)	42.5 (39.8, 45.3)	40.0 (36.6, 42.6)	39.8 (37.0, 42.7)	38.8 (36.1, 41.6)	41.4 (38.6, 44.1)	47.4 (44.3, 50.6)	43.7 (40.9, 46.7)	45.0 (42.1, 48.1)	41.9 (38.7, 45.1)
Territories	41.2 (37.9, 44.5)	42.2 (39.0, 45.5)	36.7 (33.7, 39.8)	39.6 (36.3, 43.0)	39.3 (36.2, 42.6)	37.4 (34.3, 40.6)	39.2 (35.5, 43.1)	41.6 (38.5, 44.8)	─	─
**Residency area**										
Rural	43.8 (42.6, 45.1)	43.8 (42.5, 45.2)	42.5 (41.2, 43.8)	42.2 (40.8, 43.7)	42.8 (41.5, 44.1)	42.4 (41.0, 43.8)	48.0 (46.7, 49.3)	47.5 (46.2, 48.8)	46.7 (45.5, 48.0)	46.1 (44.8, 47.4)
Urban	37.2 (36.4, 38.0)	36.9 (36.0, 37.7)	36.3 (35.5, 37.2)	36.7 (35.9, 37.6)	36.5 (35.7, 37.4)	36.7 (35.8, 37.5)	41.0 (40.2, 41.9)	40.4 (39.6, 41.3)	40.6 (39.8, 41.4)	40.7 (39.8, 41.6)
**Population centre size**										
Rural area	42.5 (41.9, 45.0)	43.1 (41.5, 44.7)	42.5 (41.0, 44.0)	42.0 (40.4, 43.7)	42.5 (41.0, 44.0)	42.6 (41.0, 44.2)	48.5 (46.9, 50.0)	47.5 (46.0, 49.0)	47.1 (45.6, 48.6)	45.9 (44.4, 47.5)
Urban core	36.6 (35.8, 37.5)	36.1 (35.2, 37.1)	35.6 (34.7, 36.5)	35.9 (34.9, 36.8)	36.0 (35.0, 36.9)	36.2 (35.2, 37.1)	40.6 (39.7, 41.6)	39.8 (38.9, 40.7)	39.8 (39.0, 40.7)	40.2 (39.3, 41.2)
Urban fringe	40.2 (36.1, 44.0)	40.7 (35.6, 46.0)	41.1 (36.5, 45.8)	41.0 (36.3, 45.8)	38.8 (34.1, 43.7)	38.8 (33.8, 44.1)	39.3 (34.7, 44.2)	48.4 (44.2, 52.6)	46.4 (42.8, 50.1)	44.8 (41.0, 48.6)
Urban O/S CMA	41.9 (39.3, 44.5)	41.2 (38.8, 43.6)	41.0 (38.7, 43.4)	42.9 (40.6, 45.4)	41.9 (39.7, 44.1)	41.1 (38.8, 43.5)	45.7 (43.6, 47.9)	44.0 (42.1, 46.0)	44.1 (42.0, 46.2)	42.9 (40.7, 45.2)
Secondary urban core	38.1 (32.7, 43.9)	40.9 (35.8, 46.2)	38.2 (33.0, 43.8)	40.7 (34.4, 47.2)	37.0 (32.3, 41.9)	39.0 (34.1, 44.2)	44.1 (39.5, 48.7)	42.5 (38.0, 47.2)	46.1 (42.3, 50.0)	43.8 (39.7, 47.9)
Mix of Urban/rural	42.4 (40.5, 44.2)	43.4 (41.1, 45.8)	41.5 (39.5, 43.5)	41.7 (39.6, 43.9)	42.8 (40.5, 45.2)	39.9 (37.5, 42.3)	47.3 (44.9, 49.7)	47.5 (45.1, 49.8)	46.3 (44.1, 48.4)	46.4 (44.1, 48.7)

P: point estimate; 95%CI: 95% confidence interval; AB: Alberta; BC: British Columbia; MB: Manitoba; NB: New Brunswick; NL: Newfoundland and Labrador; NS: Nova Scotia; ON: Ontario; PEI: Prince Edward Island; QC: Quebec; SK: Saskatchewan; Urban O/S CMA: Urban center outside census metropolitan area.

Table 3 shows the point estimates of a higher level of life satisfaction (score 9 or 10) for different population groups standardized to the age and gender composition of the 2018 Canadian population. Smaller CIs were generally evident. Thus, the increased high level of life satisfaction cannot be ascribed to a growth in the Canadian population or different age- or gender- population distributions. At the national level, age and gender standardized high life satisfaction slightly increased by 2.6% from 2009 to 2018. This is consistent with the fact that the average life dissatisfaction score declined from 13.9% in 2009 to 12.7% in 2018 (Table S2). The details could be found in Figs. S5 and S6. Residents in the Atlantic Provinces of Newfoundland & Labrador, Prince Edward Island, and the Prairie Province of Saskatchewan reported higher levels of satisfied life and lower levels of dissatisfied life compared to residents living in other provinces across all time points (Fig. S7,S8). Likewise, the standardized life satisfaction in rural residents was also consistently higher than their urban counterparts.

**Table 3:** Age-and gender- standardized annual life satisfaction scored nine and ten, Canada, 2009–2018.

Variable	2009	2010	2011	2012	2013	2014	2015	2016	2017	2018
	P% (95%CI)	P% (95%CI)	P% (95%CI)	P% (95%CI)	P% (95%CI)	P% (95%CI)	P% (95%CI)	P% (95%CI)	P% (95%CI)	P% (95%CI)
National level	39.0 (38.6, 39.4)	38.7 (38.3, 39.1)	38.6 (38.2, 39.0)	39.0 (38.6, 39.4)	39.3 (38.9, 39.6)	39.0 (38.6, 39.4)	42.0 (41.6, 42.4)	41.7 (41.3, 42.2)	41.6 (41.2, 42.0)	41.6 (40.9, 42.4)
**Province of residence**										
AB	37.6 (36.3, 38.9)	37.3 (36.0, 38.6)	38.8 (37.5, 40.1)	38.2 (36.9, 39.5)	38.7 (37.5, 40.0)	38.5 (37.3, 39.8)	42.2 (40.9, 43.5)	40.1 (38.9, 41.3)	40.0 (38.8, 41.1)	39.5 (38.3, 40.7)
BC	37.7 (36.6, 38.9)	35.8 (34.7, 36.9)	37.3 (36.2, 38.4)	35.6 (34.5, 36.7)	38.4 (37.3, 39.5)	37.4 (36.3, 38.5)	39.7 (38.5, 40.8)	40.3 (39.1, 41.4)	38.7 (37.6, 39.7)	39.7 (38.6, 40.9)
MB	38.5 (36.8, 40.2)	38.1 (36.4, 39.8)	38.2 (36.5, 39.9)	40.2 (38.5, 41.8)	39.0 (37.4, 40.6)	40.6 (39.0, 42.3)	41.8 (39.9, 43.7)	39.9 (38.1, 41.8)	42.7 (40.9, 44.6)	41.2 (39.4, 43.1)
NB	44.6 (42.8, 46.9)	45.2 (43.1, 47.2)	40.8 (38.8, 42.9)	38.6 (37.7, 39.5)	41.4 (39.4, 43.5)	42.6 (40.6, 44.6)	43.0 (40.4, 45.5)	44.7 (42.3, 47.0)	45.8 (43.5, 48.2)	42.2 (41.3, 43.1)
NL	45.4 (43.1, 47.7)	44.8 (42.5, 47.1)	44.4 (42.1, 46.7)	42.4 (40.0, 44.7)	47.8 (45.5, 50.1)	43.0 (40.7, 44.4)	47.7 (45.2, 50.2)	49.3 (46.9, 51.8)	44.0 (41.6, 46.5)	42.6 (40.0, 45.0)
NS	42.4 (40.4, 44.4)	43.1 (41.0, 45.1)	40.9 (38.9, 42.9)	41.6 (39.6, 43.6)	40.5 (38.5, 42.4)	40.0 (38.1, 42.0)	43.2 (41.1, 45.2)	42.5 (40.6, 44.5)	41.4 (39.4, 43.4)	40.8 (38.8, 42.8)
ON	37.9 (37.2, 38.5)	38.1 (37.4, 38.8)	38.3 (37.7, 39.0)	39.3 (38.6, 40.0)	39.8 (39.1, 40.5)	39.5 (38.8, 40.1)	41.8 (41.1, 42.6)	41.8 (41.0, 42.5)	42.0 (41.3, 42.7)	41.4 (40.6, 42.2)
PEI	45.0 (41.7, 48.3)	44.5 (41.4, 47.7)	41.2 (37.9, 44.5)	39.2 (37.1, 41.3)	40.3 (38.5, 42.4)	41.2 (38.0, 44.4)	45.7 (42.3, 49.0)	43.6 (40.4, 46.9)	42.5 (39.4, 45.7)	39.7 (36.6, 42.8)
QC	38.9 (38.0, 39.8)	38.2 (37.3, 39.1)	38.2 (37.3, 39.1)	38.6 (37.7, 39.5)	36.9 (36.1, 37.8)	36.7 (35.9, 37.6)	41.4 (40.4, 42.3)	41.7 (40.8, 42.6)	41.8 (41.0, 42.7)	42.2 (41.3, 43.1)
SK	39.5 (37.9, 41.1)	40.3 (38.7, 42.0)	39.8 (38.1, 41.4)	41.0 (39.3, 42.6)	39.6 (38.0, 41.2)	41.1 (39.4, 42.7)	45.9 (43.9, 48.0)	44.1 (42.1, 46.1)	44.1 (42.1, 46.1)	40.6 (38.6, 42.6)
Territories	37.9 (34.9, 41.0)	40.0 (37.1, 42.9)	34.4 (31.6, 37.1)	39.8 (36.6, 43.0)	38.4 (35.5, 41.3)	36.2 (33.5, 38.8)	41.4 (38.1, 44.8)	40.7 (37.8, 43.5)	-	-
**Residency area**										
Rural	42.9 (42.1, 43.6)	42.6 (41.8, 43.3)	41.8 (41.0, 42.5)	42.1 (41.3, 42.8)	42.8 (42.1, 43.6)	42.2 (41.5, 43.0)	45.9 (45.0, 46.7)	46.4 (45.6, 47.2)	45.9 (45.1, 46.7)	45.1 (44.3, 45.9)
Urban	37.5 (37.1, 38.0)	37.3 (36.8, 37.7)	37.5 (37.1, 38.0)	37.9 (37.5, 38.4)	37.9 (37.5, 38.4)	37.8 (37.4, 38.3)	40.6 (40.1, 41.1)	40.2 (39.7, 40.7)	40.1 (39.6, 40.6)	39.8 (39.4, 40.3)
**Population centre size**										
Rural area	42.3 (41.3, 43.3)	42.3 (41.4, 43.2)	42.1 (42.1, 41.2)	42.3 (41.4, 43.2)	42.8 (42.9, 42.0)	42.5 (42.4, 41.5)	46.0 (45.0, 47.0)	46.4 (45.4, 47.3)	45.8 (44.8, 46.8)	44.5 (43.5, 45.5)
Urban core	36.7 (36.2, 37.3)	36.3 (35.8, 36.8)	36.6 (36.5, 36.0)	36.9 (36.3, 37.4)	37.3 (37.2, 36.7)	37.3 (37.0, 36.5)	40.1 (39.6, 40.7)	39.7 (39.1, 40.2)	39.4 (38.9, 39.9)	39.3 (38.7, 39.8)
Urban fringe	40.1 (37.4, 42.8)	39.2 (36.5, 41.9)	43.0 (42.8, 40.1)	40.0 (37.3, 42.7)	39.7 (39.5, 36.8)	39.9 (39.2, 36.4)	39.6 (36.8, 42.3)	45.7 (43.0, 48.3)	44.4 (42.1, 46.7)	44.2 (41.8, 46.6)
Urban O/S CMA	40.3 (39.0, 41.7)	40.4 (39.1, 41.7)	40.3 (40.2, 38.9)	41.6 (40.2, 42.9)	41.5 (41.1, 39.9)	41.4 (41.2, 40.0)	43.5 (42.1, 44.8)	42.1 (40.9, 43.4)	42.8 (41.4, 44.1)	41.8 (40.4, 43.2)
Secondary urban core	35.3 (31.8, 38.8)	38.9 (35.5, 42.2)	38.4 (38.4, 34.9)	37.2 (33.8, 40.6)	38.8 (38.3, 35.5)	40.0 (39.7, 36.8)	42.8 (39.7, 46.0)	40.6 (37.7, 43.5)	43.3 (40.6, 46.0)	40.5 (37.9, 43.1)
Mix of Urban/rural	42.8 (41.8, 43.8)	42.2 (41.2, 43.3)	41.1 (41.0, 39.9)	41.6 (40.5, 42.7)	42.1 (41.9, 40.6)	40.8 (40.7, 39.5)	45.4 (43.9, 47.0)	46.4 (45.0, 47.9)	46.4 (45.0, 47.8)	46.5 (45.1, 48.0)

P: point estimate; 95%CI: 95% confidence interval; AB: Alberta; BC: British Columbia; MB: Manitoba; NB: New Brunswick; NL: Newfoundland and Labrador; NS: Nova Scotia; ON: Ontario; PEI: Prince Edward Island; QC: Quebec; SK: Saskatchewan; Urban O/S CMA: Urban center outside census metropolitan area.

### Population-based social support trends and their association with life satisfaction

Data on social support were not available for all the provinces over the study period. The information on data availabilities of social support is documented in Table S3. During 2009 and 2010, the proportion of the Canadian population reporting a high level of social support increased from 30.2 to 32.9%; from 2011 to 2018, the average population score for social support increased slightly between 2012 and 2014, then decreased in 2015 and remained stable until 2018 (see Table S4). Both men and women showed increasing proportions reporting high levels of social support between 2011 and 2018, with women having a higher proportion reporting a higher level of social support compared to men. The proportion reporting a high level of social support decreased with age with those aged 70 + reporting the smallest proportion of high social support. Those who lived in the urban fringe and secondary urban cores reported larger proportions with high social support (Table S5).

**Figure 2:**
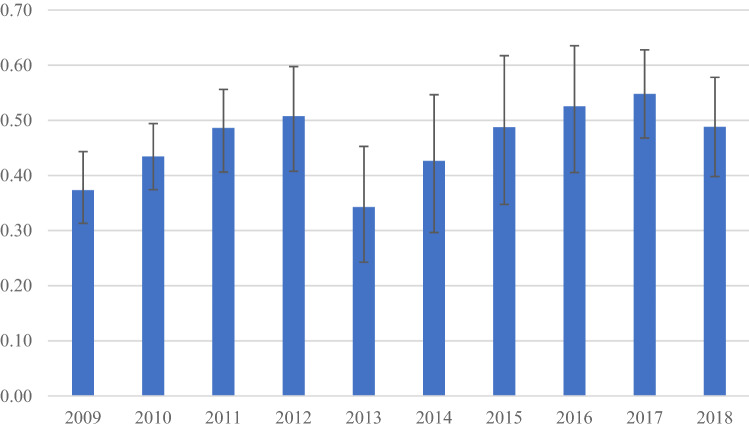
Correlations between life satisfaction and social support, Canada, 2009–2018. Note: age, gender, province, type of residential areas, population size, marital status, educational attainment, total household income, immigrant status, self-perceived health, self-rated stress, physical activity, chronic conditions, type of smokers, and type of drinkers were controlled.

Table 4 and Fig. 2 illustrate the associations between social support and life satisfaction ranging from 2009 to 2018. Social support and life satisfaction were positively correlated after controlling for *age, gender, province of residence, residential area type, population centre size, marital status, the highest education level, total household income, immigrant status, self-perceived health, self-rated stress, physical activity, chronic conditions, type of smoker,* and *type of drinker*. The strength of the association between social support and life satisfaction varied across the study period, increasing from 0.37 in 2009 to 0.51 in 2012, and then decreasing significantly in 2013 before a steady increase in the next following years with a slight decline in 2018. Women had a larger increase in this correlation during the study period than men (0.07 versus 0.16). We also observed mixed patterns in this association for different age groups, but this was more obvious for those in their thirties. People aged 70 + had the smallest variation in terms of correlation between social support and life satisfaction. People living in urban and rural regions had different correlations between social support and life satisfaction. Rural areas, urban core regions, and urban center outside CMA experienced an increase in the correlation between social support and life satisfaction over time. The rural–urban gap in terms of the relationship between social support and life satisfaction widened over time from 2010 to 2018.

**Table 4:** Multivariate regression analyses between life satisfaction and social support, Canada, 2009–2018.

Variable	2009	2010	2011	2012	2013	2014	2015	2016	2017	2018
	Coef (95% CI)	Coef (95% CI)	Coef (95% CI)	Coef (95% CI)	Coef (95% CI)	Coef (95% CI)	Coef (95% CI)	Coef (95% CI)	Coef (95% CI)	Coef (95% CI)
National level	0.37 (0.31, 0.44)	0.43 (0.37, 0.49)	0.49 (0.41, 0.56)	0.51 (0.41, 0.60)	0.34 (0.24, 0.45)	0.43 (0.30, 0.55)	0.49 (0.35, 0.62)	0.53 (0.41, 0.64)	0.55 (0.47, 0.63)	0.49 (0.40, 0.58)
**Gender**										
Men	0.38 (0.29, 0.46)	0.44 (0.35, 0.53)	0.50 (0.38, 0.62)	0.52 (0.39, 0.65)	0.31 (0.18, 0.44)	0.47 (0.31, 0.64)	0.40 (0.22, 0.57)	0.48 (0.33, 0.63)	0.60 (0.49, 0.72)	0.45 (0.32, 0.58)
Women	0.36 (0.27, 0.45)	0.43 (0.35, 0.51)	0.45 (0.35, 0.55)	0.48 (0.34, 0.61)	0.35 (0.20, 0.51)	0.34 (0.15, 0.53)	0.58 (0.38, 0.78)	0.56 (0.40, 0.72)	0.47 (0.36, 0.58)	0.52 (0.40, 0.64)
**Age (years)**										
12–19	0.25 (0.05, 0.46)	0.36 (0.21, 0.51)	0.44 (0.25, 0.62)	0.58 (0.30, 0.85)	0.32 (−0.01, 0.65)	0.29 (−0.03, 0.60)	0.21 (−0.07, 0.48)	0.60 (0.30, 0.90)	0.54 (0.31, 0.78)	0.40 (0.15, 0.66)
20–29	0.29 (0.15, 0.43)	0.36 (0.24, 0.49)	0.38 (0.21, 0.56)	0.35 (0.04, 0.66)	0.39 (0.14, 0.64)	0.50 (−0.06, 1.05)	0.61 (0.29, 0.92)	0.72 (0.44, 1.00)	0.46 (0.24, 0.67)	0.50 (0.22, 0.78)
30–39	0.47 (0.32, 0.62)	0.51 (0.37, 0.66)	0.66 (0.44, 0.88)	0.76 (0.51, 1.01)	0.44 (0.16, 0.73)	0.59 (0.20, 0.98)	0.44 (0.20, 0.67)	0.50 (0.28, 0.73)	0.63 (0.42, 0.84)	0.56 (0.36, 0.76)
40–49	0.34 (0.17, 0.52)	0.35 (0.17, 0.53)	0.55 (0.33, 0.76)	0.55 (0.30, 0.80)	0.33 (−0.04, 0.70)	0.50 (0.11, 0.89)	0.50 (0.18, 0.82)	0.38 (0.09, 0.67)	0.49 (0.32, 0.67)	0.43 (0.19, 0.67)
50–59	0.40 (0.27, 0.53)	0.53 (0.38, 0.68)	0.59 (0.42, 0.75)	0.52 (0.33, 0.72)	0.34 (0.09, 0.60)	0.44 (0.23, 0.66)	0.61 (0.22, 1.01)	0.52 (0.26, 0.78)	0.69 (0.47, 0.92)	0.53 (0.32, 0.75)
60–69	0.42 (0.28, 0.57)	0.41 (0.27, 0.56)	0.36 (0.17, 0.55)	0.35 (0.17, 0.53)	0.21 (0.01, 0.42)	0.37 (0.16, 0.58)	0.28 (−0.03, 0.58)	0.46 (0.24, 0.69)	0.43 (0.21, 0.64)	0.51 (0.32, 0.70)
70 +	0.36 (0.18, 0.55)	0.44 (0.27, 0.62)	0.36 (0.20, 0.52)	0.39 (0.17, 0.61)	0.25 (0.06, 0.45)	0.20 (0.01, 0.39)	0.48 (0.11, 0.84)	0.43 (0.18, 0.68)	0.54 (0.36, 0.72)	0.35 (0.15, 0.56)
**Province of residence**										
AB	–	–	–	–	–	–	0.48 (0.34, 0.63)	0.53 (0.42, 0.65)	0.60 (0.49, 0.72)	0.55 (0.41, 0.68)
BC	0.33 (0.20, 0.45)	0.48 (0.37, 0.60)	0.60 (0.48, 0.73)	0.60 (0.44, 0.76)	–	–	–	–	0.53 (0.41, 0.66)	0.42 (0.29, 0.56)
MB	–	–	–	–	–	–	–	–	–	–
NB	0.37 (0.21, 0.53)	0.41 (0.24, 0.58)	–	–	–	–	–	–	–	–
NL	–	–	–	–	–	–	–	–	0.14 (−0.10, 0.38)	0.45 (0.20, 0.71)
NS	–	–	–	–	0.49 (0.24, 0.74)	0.66 (0.37, 0.94)	–	–	–	–
ON	–	–	–	–	–	–	–	–	–	–
PEI	–	–	–	–	–	–	0.37 (0.08, 0.66)	0.22 (−0.07, 0.51)	0.53 (0.22, 0.84)	0.74 (0.44, 1.04)
QC	0.40 (0.32, 0.49)	0.39 (0.31, 0.47)	0.41 (0.31, 0.50)	0.41 (0.29, 0.53)	0.32 (0.21, 0.44)	0.40 (0.27, 0.53)	–	–	–	–
SK	0.28 (0.15, 0.42)	0.44 (0.28, 0.61)	–	–	–	–	–	–	–	–
Territories	0.19 (−0.07, 0.46)	0.34 (−0.13, 0.80)	0.64 (0.44, 0.83)	0.65 (0.38, 0.91)	–	–	0.46 (−0.07, 0.98)	0.55 (0.14, 0.96)	–	–
**Residency area**										
Rural	0.38 (0.27, 0.48)	0.48 (0.36, 0.60)	0.52 (0.43, 0.61)	0.59 (0.48, 0.70)	0.38 (0.26, 0.51)	0.45 (0.30, 0.60)	0.31 (0.01, 0.61)	0.31 (0.10, 0.51)	0.46 (0.31, 0.62)	0.38 (0.24, 0.51)
Urban	0.37 (0.29, 0.44)	0.42 (0.35, 0.49)	0.30 (0.15, 0.45)	0.17 (0.03, 0.32)	0.19 (0.03, 0.36)	0.37 (0.21, 0.54)	0.50 (0.35, 0.65)	0.55 (0.43, 0.68)	0.56 (0.47, 0.65)	0.51 (0.41, 0.61)
**Population centre size**										
Rural area	0.35 (0.00, 0.71)	0.43 (0.36, 0.51)	0.53 (0.43, 0.62)	0.59 (0.47, 0.71)	0.38 (0.23, 0.52)	0.44 (0.27, 0.61)	0.24 (−0.11, 0.60)	0.28 (0.04, 0.52)	0.44 (0.25, 0.64)	0.50 (0.33, 0.67)
Urban core	0.35 (0.27, 0.44)	0.35 (−0.08, 0.78)	0.71 (−0.02, 1.44)	0.52 (0.02, 1.02)	0.37 (−0.33, 1.07)	0.04 (−0.40, 0.48)	0.51 (0.34, 0.67)	0.53 (0.39, 0.67)	0.58 (0.49, 0.68)	0.55 (0.44, 0.66)
Urban fringe	–	–	–	–	–	–	0.23 (−0.67, 1.13)	0.68 (0.08, 1.29)	0.24 (−0.25, 0.73)	−0.10 (−0.52, 0.33)
Urban O/S CMA	0.26 (0.04, 0.49)	0.42 (0.25, 0.59)	0.44 (0.19, 0.69)	0.59 (0.27, 0.91)	0.30 (0.01, 0.60)	0.55 (0.21, 0.89)	0.54 (0.16, 0.91)	0.72 (0.39, 1.05)	0.30 (0.12, 0.48)	0.49 (0.12, 0.86)
Secondary urban core	–	–	–	–	–	–	0.54 (−0.14, 1.22)	0.52 (−0.01, 1.05)	0.43 (0.04, 0.82)	0.22 (−0.23, 0.66)
Mix of Urban/rural	0.68 (0.14, 1.23)	0.26 (−0.23, 0.74)	0.29 (−0.21, 0.79)	0.42 (−0.48, 1.32)	1.03 (0.46, 1.60)	1.01 (0.10, 1.93)	0.67 (0.17, 1.16)	0.41 (0.04, 0.79)	0.50 (0.22, 0.78)	0.22 (−0.03, 0.46)

95%CI: 95% confidence interval; AB: Alberta; BC: British Columbia; MB: Manitoba; NB: New Brunswick; NL: Newfoundland and Labrador; NS: Nova Scotia; ON: Ontario; PEI: Prince Edward Island; QC: Quebec; SK: Saskatchewan; Urban O/S CMA: Urban center outside census metropolitan area.

## Discussion

The present study examined the trends and patterns of life satisfaction at a national level and explored changes in the associations between social support and life satisfaction between 2009 and 2018. We observed a gradual increase in average annual life satisfaction score from 8.01 in 2009 to 8.12 in 2018 and the increase largely occurred among residents living in the provinces of Ontario, British Columbia, and Quebec. Women, between 12 and 19 years of age, living in rural areas, were more likely to report having a satisfied life. Based on the available data on social support, we observed a positive correlation between social support and life satisfaction over time. This finding should be interpreted with caution given it came from selected provinces and study years.

Empirical studies have consistently reported that life satisfaction varies widely among countries, as GDP per capita, social support, healthy life expectancy, freedom to make life choices, and generosity, contribute to variations in life satisfaction among countries globally^31,32^. High-income countries tend to have higher average life satisfaction scores, and most countries that have experienced sustained economic growth and sociopolitical stability have seen increasing life satisfaction levels^33^. Canada has experienced rapid population growth and growing social equality in recent decades, which is consistent with a rising trend of average life satisfaction score^34^.

We also found that people living in CMAs and Census Agglomerations with populations more than 50,000 had a lower level of life satisfaction than those who lived in small cities, towns, and rural areas of the rest of the country. This was also reported in a recent study by Helliwell et al.^35^ covering the period 2009 to 2014 using a different combination of national survey datasets. Other studies from high-income countries, i.e., the United States, Australia, the United Kingdom, and continental Europe, reported similar findings^11,36–38^. Our current study observed that people from different provinces of Canada report different levels of life satisfaction. Throughout the study period, the Atlantic provinces of Canada showed small variations in life satisfaction, and together with Saskatchewan, they report higher levels of life satisfaction. Sharpe et al.^39^ suggested that a sense of belonging to local communities, which was generally higher in Atlantic Canada, small CMAs, and rural areas, explained some of the geographical variations in life satisfaction. Residents benefit from enjoying more safety and freedom and engaging more with family and friends, and community activities rather than material pursuits^40^.

The gender and age differences in life satisfaction observed in the present study are consistent with what has been identified in the literature^14^. Women were generally more satisfied with their lives compared to men although this gap shrinks with age. A study by Blanchflower and Oswald^41^ compared over 20,000 individuals from both the USA and Britain and found that women reported higher levels of life satisfaction in both countries. Likewise, the average life satisfaction score in women was higher than men among the populations of Sweden, Austria, and Germany aged 50 and older^42^. While this is true for high-income countries, there is limited evidence of this effect in low-income countries^43,44^. Cultural and social norms may be one of the major elements that play an important role in subjective well-being^45,46^. In general, women have larger and more varied social networks with more friends and more social support than men, which might in turn help to remain and promote a higher level of satisfaction^47^. Stevenson and Wolfers^48^ also found that women were more satisfied than men in the United States and Western Europe, but this gap had declined in recent years. The decrease in gender difference in life satisfaction could partially be explained by relative declines in life satisfaction among women compared to men counterparts over time. In comparison with men, women might find the complexity and increased pressure in their modern lives to have come at some cost to their happiness^48^.

As expected, we found that young and older people were more satisfied with their lives than middle-aged people, which is consistent with previous findings^49,50^. This finding reflects the differential impact of key and major life events that happened at different stages of life (marriage, giving birth, employment, etc.). Midlife crisis, centering on major life disruptions seen as typical to this life stage, such as job loss, divorce, or the death of parents, has been found to be significantly correlated with a lower level of life satisfaction, whereas people become more satisfied after they retired^51,52^.

Social support plays an important role in shaping life satisfaction^53^. It is an external resource to cope with psychological tension(s) by improving social adaptability and being more resilient to adverse environmental conditions^54^. In line with the literature on social support, a positive correlation between social support and life satisfaction was observed^55,56^. More importantly, we observed that an increase in the level of social support was positively correlated with a steady increase in life satisfaction over time. Although the repeated cross-sectional study design cannot draw a direct causal relationship between social support and life satisfaction, our findings corroborate the literature by illustrating its variations linked with changes in life satisfaction over time. These finding sheds light on public health promotion to advocate social support at a population level.

In the present study, there were variations in terms of data on social support among provinces of Canada in a particular year. Notably, the correlation between social support and life satisfaction in 2013 was substantially different from the rest of the studied period, as only two provinces—Nova Scotia and Quebec collected data on social support. Since 2014, the correlations between social support and life satisfaction had steadily increased. Due to the constraint of data on social support across different years in different provinces, we cannot identify any specific temporal pattern for the relationship between social support and life satisfaction in various Canadian population subgroups.

## Limitations

There are several limitations to be noted. First, although data analyzed were from a series of large national, representative surveys, data on social support varied between surveys and were only available for selected provinces and territories in different years. Thus, comparisons on social support and its correlation with life satisfaction cannot be made directly. Second, information on social support and life satisfaction was self-reported, which is subject to recall bias and measurement errors. However, the single item self-reported life satisfaction has been proven to be stable and reliable^57^. Similarly, measurements on social support are also from standard validated questionnaires^24,28^. Third, this secondary analysis was based on a series of cross-sectional national surveys. We examined the strength of correlations between social support and life satisfaction over time. Although data were cross-sectional, the series of national surveys provide insights into dynamic changes in life satisfaction and how they were correlated with social support.

## Conclusions

Overall, the level of life satisfaction in Canada has gradually increased from 2009 to 2018, and differences in life satisfaction were observed in sociodemographic groups. A positive relationship between social support and life satisfaction was consistently observed for the provinces and the study years for which data were available. The findings of the present study suggest that strengthening social support could be the target for public health promotion aimed at improving life satisfaction at a population level. Future research is needed to further explore health and social inequities among different population subgroups that may be reflected in their various levels of life satisfaction.

## Supplementary Information

Supplementary Information.

## Data Availability

This research was conducted in part at the Saskatchewan Research Data Centre, a part of the Canadian Research Data Centre Network (CRDCN). This service is provided through the support of the University of Saskatchewan, the Canadian Foundation for Innovation, the Canadian Institutes of Health Research, the Social Science and Humanity Research Council, and Statistics Canada. This research was also conducted in part at the Quebec Interuniversity Centre for Social Statistics (QICSS) at McGill University, part of the Canadian Research Data Centre Network (CRDCN). This service is provided through the support of QICSS’ Member Universities, the province of Quebec, the Canadian Foundation for Innovation, the Canadian Institutes of Health Research, the Social Science and Humanity Research Council, the Fonds du Recherche du Québec (Nature et Technologie, Santé, Société et Culture), and Statistics Canada.
