# Supplementary material for: Trends and patterns of life satisfaction and its relationship with social support in Canada, 2009 to 2018
Source: Sci Rep. 2022 Jun 12;12:9720. doi: 10.1038/s41598-022-13794-x (PMC9189104; doi:10.1038/s41598-022-13794-x)
Supplement: Supplementary file 1 — Supplementary Information. [file 41598_2022_13794_MOESM1_ESM.docx]

| **Table S1** The annual life satisfaction scored six or less, Canada, 2009-2018 | | | | | | | | | | |
| --- | --- | --- | --- | --- | --- | --- | --- | --- | --- | --- |
| Variable | 2009 | 2010 | 2011 | 2012 | 2013 | 2014 | 2015 | 2016 | 2017 | 2018 |
|  | P%  (95%CI) | P%  (95%CI) | P%  (95%CI) | P%  (95%CI) | P%  (95%CI) | P%  (95%CI) | P%  (95%CI) | P%  (95%CI) | P%  (95%CI) | P%  (95%CI) |
| National level | 12.4  (12.0, 12.9) | 12.6  (12.1, 13.1) | 12.4  (11.9, 12.9) | 12.6  (12.1, 13.2) | 12.7  (12.2, 13.3) | 12.6  (12.0, 13.1) | 11.3  (10.8, 11.8) | 11.8  (11.3, 12.3) | 11.6  (11.2, 12.1) | 11.3  (10.9, 11.8) |
| Gender |  |  |  |  |  |  |  |  |  |  |
| Men | 12.3  (11.6, 13.0) | 12.9  (12.1, 13.7) | 12.4  (11.6, 13.1) | 12.5  (11.7, 13.3) | 12.3  (11.5, 13.0) | 12.5  (11.7, 13.3) | 10.8  (10.2, 11.5) | 11.6  (10.9, 12.4) | 11.7  (11.0, 12.4) | 10.8  (10.1, 11.5) |
| Women | 12.5  (11.9, 13.2) | 12.3  (11.7, 13.0) | 12.5  (12.2, 13.6) | 12.8  (12.0, 13.5) | 13.2  (12.4, 14.0) | 12.6  (12.4, 13.8) | 11.8  (11.1, 12.5) | 12.0  (11.3, 12.6) | 11.6  (11.0, 12.2) | 11.9  (11.2, 12.8) |
| Age (years) |  |  |  |  |  |  |  |  |  |  |
| 12-19 | 6.8  (2.9, 7.8) | 6.6  (5.8, 7.5) | 6.7  (5.8, 7.7) | 6.4  (5.4, 7.5) | 6.8  (5.9, 7.8) | 6.5  (5.6, 7.5) | 4.9  (4.1, 5.9) | 6.4  (5.2, 7.8) | 5.3  (4.3, 6.4) | 6.3  (5.2, 7.6) |
| 20-29 | 10.2  (9.2, 11.4) | 9.1  (8.2, 10.1) | 10.0  (8.9, 11.2) | 10.8  (9.5, 12.3) | 9.9  (8.8, 11.1) | 10.3  (9.1, 11.5) | 8.4  (7.3, 9.6) | 10.0  (8.6, 11.5) | 10.3  (9.1, 11.6) | 9.5  (8.3, 10.9) |
| 30-39 | 10.7  (9.6, 11.8) | 11.7  (10.5, 13.1) | 10.8  (9.7, 12.0) | 11.0  (9.7, 12.3) | 11.2  (10.0, 12.6) | 12.1  (10.7, 13.7) | 9.8  (8.7, 10.9) | 9.4  (8.5, 10.5) | 10.4  (9.4, 11.5) | 10.0  (8.9, 11.2) |
| 40-49 | 13.8  (12.5, 15.1) | 14.8  (13.2, 16.6) | 14.3  (12.8, 15.9) | 14.5  (12.9, 16.3) | 15.2  (13.5, 17.1) | 13.4  (12.0, 15.0) | 12.8  (11.4, 14.3) | 12.2  (11.0, 13.5) | 12.3  (11.2, 13.5) | 13.5  (12.0, 15.2) |
| 50-59 | 14.7  (13.5, 16.0) | 14.9  (13.5, 16.4) | 14.4  (13.0, 16.0) | 14.2  (12.7, 15.8) | 15.3  (13.7, 17.1) | 14.3  (12.8, 15.8) | 13.5  (12.3, 14.8) | 14.1  (12.9, 15.4) | 14.1  (12.7, 15.6) | 13.1  (11.8, 14.4) |
| 60-69 | 14.4  (13.2, 15.8) | 13.4  (12.3, 14.6) | 14.7  (13.3, 16.2) | 14.2  (12.9, 15.6) | 13.8  (12.6, 15.2) | 14.0  (12.9, 15.1) | 13.0  (11.8, 14.4) | 13.7  (12.4, 15.06) | 13.5  (12.4, 14.7) | 12.0  (11.0, 13.1) |
| 70+ | 16.9  (15.8, 18.0) | 17.8  (16.7, 19.1) | 15.9  (14.7, 17.1) | 17.1  (15.9, 18.3) | 15.9  (14.8, 17.0) | 16.8  (15.7, 18.0) | 16.2  (14.9, 17.5) | 16.4  (15.1, 17.9) | 14.0  (12.9, 15.2) | 13.6  (12.6, 14.7) |
| Province of residence |  |  |  |  |  |  |  |  |  |  |
| AB | 13.0  (11.5, 14.6) | 13.2  (117, 15.0) | 12.8  (11.4, 14.5) | 13.0  (11.3, 14.9) | 12.0  (10.2, 14.0) | 11.7  (10.4, 13.2) | 10.4  (9.3, 11.6) | 11.7  (10.6, 12.9) | 12.4  (11.4, 13.6) | 12.0  (10.8, 13.3) |
| BC | 14.1  (12.9, 15.4) | 14.0  (12.7, 15.5) | 12.8  (11.6, 14.2) | 14.9  (13.3, 16.7) | 13.4  (11.7, 15.3) | 12.9  (11.7, 14.2) | 11.9  (10.8, 13.1) | 12.7  (11.5, 13.9) | 12.9  (11.7, 14.3) | 12.8  (11.6, 14.2) |
| MB | 12.2  (10.5, 14.1) | 13.3  (11.3, 15.6) | 13.6  (11.2, 16.4) | 14.8  (12.3, 17.7) | 12.2  (13.7, 14.4) | 10.9  (9.3, 12.7) | 11.5  (9.9, 13.3) | 12.9  (11.2, 14.9) | 10.7  (8.9, 12.8) | 11.0  (9.40, 13.0) |
| NB | 11.5  (9.9, 13.2) | 12.4  (10.4, 14.7) | 11.7  (9.7, 13.9) | 10.5  (9.0, 12.3) | 11.7  (10.1, 13.6) | 12.1  (10.4, 14.1) | 13.8  (11.5, 16.4) | 11.6  (9.8, 13.7) | 10.1  (8.4, 12.1) | 11.3  (9.6, 13.4) |
| NL | 12.0  (9.8, 14.5) | 11.2  (9.5, 13.2) | 10.4  (8.6, 12.7) | 13.4  (10.8, 16.6) | 9.4  (7.7, 11.4) | 12.1  (9.9, 14.7) | 12.8  (10.4, 15.7) | 10.0  (8.2, 12.1) | 11.4  (9.5, 13.7) | 10.9  (8.8, 13.3) |
| NS | 14.0  (11.7, 16.7) | 12.0  (10.3, 14.0) | 10.5  (9.0, 12.3) | 11.1  (9.3, 13.3) | 12.8  (11.1, 14.7) | 12.5  (10.7, 14.5) | 11.8  (9.9, 14.0) | 13.0  (11.3, 15.0) | 12.7  (11.0, 14.7) | 13.8  (11.9, 16.0) |
| ON | 13.5  (12.7, 14.3) | 13.7  (12.8, 14.7) | 14.2  (13.2, 15.1) | 13.2  (12.2, 14.2) | 14.3  (13.3, 15.3) | 14.1  (13.1, 15.2) | 11.7  (10.8, 12.6) | 13.0  (12.0, 13.9) | 12.0  (11.1, 12.9) | 11.6  (10.7, 12.6) |
| PEI | 10.9  (8.5, 13.8) | 8.0  (6.0, 10.5) | 11.1  (8.5, 14.4) | 11.1  (8.7, 14.0) | 11.0  (8.7, 13.9) | 8.4  (6.5, 10.8) | 9.1  (7.1, 11.5) | 10.2  (7.9, 13.1) | 11.4  (9.0, 14.3) | 11.0  (8.7, 13.8) |
| QC | 9.5  (8.6, 10.4) | 9.9  (9.1, 10.9) | 9.5  (8.5, 10.5) | 10.3  (9.3, 11.4) | 10.8  (9.8, 11.8) | 11.0  (10.1, 12.1) | 10.5  (9.6, 11.5) | 9.7  (8.8, 10.7) | 10.0  (9.2, 10.8) | 9.7  (8.9, 10.5) |
| SK | 10.9  (9.6, 12.5) | 11.1  (9.5, 13.0) | 11.4  (9.8, 13.3) | 12.6  (10.7, 14.9) | 10.0  (8.6, 11.7) | 8.9  (7.5, 10.5) | 10.8  (9.1, 12.7) | 8.4  (7.2, 9.9) | 12.6  (10.7, 14.7) | 9.6  (8.0, 11.5) |
| Territories | 14.4  (12.3, 16.8) | 13.9  (11.7, 16.5) | 13.8  (11.8, 16.1) | 14.9  (12.5, 17.5) | 14.6  (12.5, 16.9) | 15.8  (15.5, 18.4) | 14.3  (11.9, 17.2) | 14.8  (12.7, 17.2) | ─ | ─ |
| Residency area |  |  |  |  |  |  |  |  |  |  |
| Urban | 12.8  (12.2, 13.3) | 13.1  (12.5, 13.7) | 12.9  (12.3, 13.5) | 13.3  (12.7, 14.0) | 13.2  (12.5, 13.8) | 12.9  (12.3, 13.6) | 11.8  (11.2, 12.3) | 12.2  (11.6, 12.8) | 12.1  (11.5, 12.60) | 11.4  (11.1, 12.2) |
| Rural | 10.8  (10.1, 11.5) | 10.3  (9.6, 11.1) | 10.3  (95, 11.1) | 9.6  (8.9, 10.3) | 10.7  (9.8, 11.6) | 10.9  (10.0, 11.9) | 9.2  (8.5, 9.9) | 9.9  (9.2, 10.7) | 9.6  (9.0, 10.3) | 10.0  (9.3, 10.8) |
| Population centre size |  |  |  |  |  |  |  |  |  |  |
| Rural area | 10.4  (9.5, 11.3) | 10.3  (9.4, 11.2) | 10.2  (9.3, 11.2) | 9.0  (8.2, 9.8) | 11.0  (10.0, 12.1) | 10.8  (9.7, 11.9) | 9.4  (8.7, 10.3) | 10.2  (9.4, 11.1) | 10.6  (9.8, 11.4) | 10.5  (9.7, 11.4) |
| Urban core | 13.2  (12.6, 13.8) | 13.2  (12.5, 13.9) | 13.1  (12.4, 13.8) | 13.7  (13.0, 14.5) | 13.2  (12.5, 13.9) | 13.3  (12.7, 14.0) | 12.0  (11.4, 12.6) | 12.3  (11.7, 12.9) | 12.4  (11.8, 13.0) | 11.9  (11.3, 12.5) |
| Urban fringe | 9.6  (7.7, 11.9) | 13.6  (9.0, 20.0) | 11.2  (8.6, 14.4) | 10.8  (8.1, 14.3) | 11.0  (8.1, 14.8) | 8.1  (6.3, 10.3) | 11.3  (8.3, 15.1) | 9.2  (7.4, 11.4) | 9.5  (7.8, 11.6) | 9.4  (7.4, 11.8) |
| Urban O/S CMA | 10.8  (9.2, 12.6) | 12.6  (11.7, 11.1) | 12.6  (11.0, 14.4) | 10.4  (9.1, 11.8) | 13.4  (12.0, 15.0) | 11.4  (10.0, 13.0) | 10.4  (9.3, 11.6) | 12.1  (10.9, 13.4) | 10.6  (9.5, 11.9) | 10.5  (9.3, 11.9) |
| Secondary urban  core | 9.5  (7.2, 12.6) | 11.2  (8.5, 14.7) | 11.0  (7.9, 15.1) | 11.8  (8.4, 16.2) | 13.6  (10.1, 18.1) | 7.9  (5.9, 10.4) | 8.6  (6.5, 11.2) | 12.5  (10.0 15.6) | 9.9  (7.8, 12.4) | 9.5  (7.5, 12.0) |
| Mix of  Urban/rural | 11.6  (10.5, 12.7) | 11.6  (9.9, 13.4) | 10.6  (9.5, 11.8) | 11.6  (10.3, 13.1) | 10.9  (9.4, 12.5) | 12.7  (11.1, 14.5) | 8.8  (7.7, 10.1) | 9.5  (8.2, 10.6) | 8.2  (7.2, 9.3) | 9.3  (8.0, 10.7) |

Note: P: point estimate; 95%CI: 95% confidence interval; AB: Alberta; BC: British Columbia; MB: Manitoba; NB: New Brunswick; NL: Newfoundland and Labrador; NS: Nova Scotia; ON: Ontario; PEI: Prince Edward Island; QC: Quebec; SK: Saskatchewan.

| **Table S2** Age-and gender- standardized annual life satisfaction scored six or less, Canada, 2009-2018 | | | | | | | | | | |
| --- | --- | --- | --- | --- | --- | --- | --- | --- | --- | --- |
| Variable | 2009 | 2010 | 2011 | 2012 | 2013 | 2014 | 2015 | 2016 | 2017 | 2018 |
|  | P%  (95%CI) | P%  (95%CI) | P%  (95%CI) | P%  (95%CI) | P%  (95%CI) | P%  (95%CI) | P%  (95%CI) | P%  (95%CI) | P%  (95%CI) | P%  (95%CI) |
| National level | 13.9  (13.6, 14.2) | 13.7  (13.4, 14.0) | 13.3  (13.0, 13.5) | 13.2  (13.0, 13.5) | 13.2  (12.9, 13.4) | 13.2  (12.9, 13.4) | 12.7  (12.4, 13.0) | 12.9  (12.7, 13.2) | 12.9  (12.6, 13.2) | 12.7  (12.4, 13.0) |
| Province of residence |  |  |  |  |  |  |  |  |  |  |
| NL | 11.6  (10.0, 13.1) | 12.7  (11.2, 14.3) | 11.7  (10.2, 13.2) | 13.9  (12.3, 15.6) | 11.5  (10.0, 12.9) | 12.5  (11.0, 14.0) | 13.4  (11.6, 15.1) | 11.3  (9.8, 12.9) | 12.5  (10.8, 14.1) | 12.4  (10.8, 14.1) |
| PEI | 13.1  (10.9, 15.3) | 10.5  (8.5, 12.4) | 12.8  (10.6, 15.1) | 12.1  (10.0, 14.3) | 12.8  (10.7, 14.9) | 10.6  (8.6, 12.6) | 11.7  (9.5, 13.8) | 11.7  (9.5, 13.8) | 12.5  (10.4, 14.8) | 12.1  (10.0, 14.2) |
| NS | 14.6  (13.1, 16.0) | 13.5  (12.1, 14.9) | 13.2  (11.8, 14.6) | 13.8  (12.4, 15.3) | 14.4  (13.0, 15.8) | 13.6  (12.2, 14.9) | 12.9  (11.5, 14.3) | 14.1  (12.7, 15.5) | 14.3  (12.9, 15.8) | 15.2  (13.7, 16.6) |
| NB | 13.4  (12.0, 14.8) | 12.9  (11.6, 14.3) | 12.3  (10.9, 13.7) | 13.1  (11.8, 14.5) | 14.0  (12.6, 15.3) | 14.0  (12.6, 15.5) | 14.0  (12.2, 15.7) | 13.7  (12.1, 15.3) | 12.8  (11.2, 14.4) | 13.3  (11.7, 14.9) |
| QC | 10.9  (10.3, 11.5) | 11.1  (10.5, 11.7) | 10.5  (10.0, 11.1) | 10.6  (10.0, 11.1) | 11.6  (10.0, 12.1) | 12.0  (11.4, 12.6) | 11.4  (10.8, 12.0) | 10.7  (10.1, 11.2) | 10.9  (10.4, 11.5) | 11.1  (10.5, 11.7) |
| ON | 14.6  (14.3, 15.3) | 14.5  (14.1, 15.0) | 14.3  (13.9, 14.8) | 13.7  (13.2, 14.2) | 13.7  (13.2, 14.1) | 13.6  (13.2, 14.1) | 13.0  (12.5, 13.6) | 13.7  (13.1, 14.2) | 13.2  (12.7, 13.7) | 13.0  (12.5, 13.6) |
| MB | 15.4  (14.1, 16.6) | 15.0  (13.7, 16.2) | 13.9  (12.7, 15.1) | 14.8  (13.6, 16.0) | 13.6  (12.5, 14.8) | 13.0  (11.9, 14.1) | 13.1  (11.8, 14.4) | 14.1  (12.8, 15.4) | 12.0  (10.8, 13.2) | 13.3  (12.0, 14.6) |
| SK | 14.2  (13.1, 15.4) | 13.4  (12.2, 14.5) | 12.8  (11.7, 13.9) | 12.6  (11.5, 13.7) | 11.9  (10.9, 13.0) | 10.9  (9.8, 11.9) | 12.5  (11.1, 13.9) | 11.4  (10.1, 12.7) | 13.6  (12.2, 15.0) | 11.3  (10.0, 12.6) |
| AB | 15.2  (14.2, 16.2) | 14.4  (13.5, 15.4) | 14.1  (13.1, 15.0) | 13.8  (12.9, 14.8) | 13.0  (12.1, 13.9) | 13.4  (12.5, 14.3) | 12.8  (11.9, 13.7) | 13.8  (12.9, 14.6) | 14.7  (13.9, 15.6) | 13.9  (13.0, 14.8) |
| BC | 14.3  (13.5, 15.2) | 14.9  (14.1, 15.7) | 13.8  (13.0, 14.6) | 14.5  (13.7, 15.3) | 14.1  (13.3, 14.8) | 14.2  (13.4, 15.0) | 13.4  (12.6, 14.2) | 13.7  (12.9, 14.5) | 13.6  (12.9, 14.4) | 13.4  (12.6, 14.2) |
| Territories | 19.1  (16.5, 21.7) | 15.7  (13.4, 18.0) | 16.2  (14.0, 18.4) | 15.3  (12.9, 17.7) | 16.3  (14.0, 18.7) | 15.1  (13.1, 17.2) | 16.1  (13.5, 18.7) | 14.9  (12.8, 17.0) | - | - |
| Residency area |  |  |  |  |  |  |  |  |  |  |
| Urban | 14.2  (13.9, 14.5) | 14.3  (13.9, 14.6) | 13.8  (13.4, 14.1) | 13.7  (13.4, 14.0) | 13.6  (13.3, 13.9) | 13.6  (13.3, 13.9) | 13.3  (12.9, 13.6) | 13.3  (13.0, 13.7) | 13.3  (13.0, 13.7) | 13.2  (12.8, 13.5) |
| Rural | 13.0  (12.5, 13.5) | 12.2  (11.7, 12.7) | 11.9  (11.4, 12.3) | 11.9  (11.4, 12.4) | 12.1  (11.6, 12.5) | 12.0  (11.5, 12.5) | 11.3  (10.8, 11.9) | 11.8  (11.3, 12.3) | 11.6  (11.1, 12.1) | 11.5  (11.0, 12.0) |
| Population centre size |  |  |  |  |  |  |  |  |  |  |
| Rural area | 12.5  (11.8, 13.1) | 11.5  (10.9, 12.1) | 11.5  (10.9, 12.1) | 11.1  (10.5, 11.6) | 11.7  (11.1, 12.2) | 11.7  (11.1, 12.2) | 11.8  (11.2, 12.5) | 12.1  (11.5, 12.8) | 12.3  (11.7, 13.0) | 11.8  (11.2, 12.5) |
| Urban core | 14.5  (14.1, 14.9) | 14.5  (14.1, 14.8) | 14.0  (13.7, 14.4) | 14.0  (13.7, 14.4) | 13.8  (13.4, 14.2) | 13.9  (13.5, 14.3) | 13.5  (13.1, 13.9) | 13.4  (13.0, 13.8) | 13.6  (13.2, 14.0) | 13.5  (13.1, 13.9) |
| Urban fringe | 13.2  (11.3, 15.0) | 13.6  (11.7, 15.5) | 11.9  (10.1, 13.7) | 12.3  (10.5, 14.2) | 10.9  (9.1, 12.6) | 11.3  (9.5, 13.1) | 12.4  (10.5, 14.3) | 11.8  (10.0, 13.5) | 12.1  (10.6, 13.6) | 10.0  (8.5, 11.4) |
| Urban O/S CMA | 12.9  (12.0, 13.9) | 14.0  (13.0, 14.9) | 13.4  (12.5, 14.3) | 12.4  (11.6, 13.3) | 13.4  (12.6, 14.3) | 12.6  (11.7, 13.4) | 12.3  (11.4, 13.7) | 13.3  (12.4, 14.1) | 12.4  (11.5, 13.4) | 12.9  (11.9, 13.8) |
| Secondary urban  core | 14.1  (11.5, 16.7) | 14.2  (11.7, 16.6) | 13.4  (10.9, 15.9) | 13.6  (11.2, 16.0) | 12.4  (10.4, 14.3) | 10.4  (8.6, 12.2) | 12.2  (10.1, 14.3) | 13.9  (11.8, 16.0) | 12.1  (10.3, 13.9) | 11.0  (9.3, 12.7) |
| Mix of Urban/rural | 14.0  (13.2, 14.7) | 13.4  (12.7, 14.1) | 12.6  (11.8, 13.3) | 13.5  (12.8, 14.3) | 13.1  (12.3, 14.0) | 13.4  (12.5, 14.3) | 10.2  (9.3, 11.2) | 11.1  (10.1, 12.0) | 10.2  (9.3, 11.0) | 10.7  (9.8, 11.6) |

Note: P: point estimate; 95%CI: 95% confidence interval; AB: Alberta; BC: British Columbia; MB: Manitoba; NB: New Brunswick; NL: Newfoundland and Labrador; NS: Nova Scotia; ON: Ontario; PEI: Prince Edward Island; QC: Quebec; SK: Saskatchewan.

|  | **Table S3** Survey data available on social support across provinces, from 2009 to 2018 | | | | | | | | | |  |
| --- | --- | --- | --- | --- | --- | --- | --- | --- | --- | --- | --- |
| Year/Provinces | Alberta | British Columbia | Manitoba | New Brunswick | Newfoundland and Labrador | Nova Scotia | Ontario | Prince Edward Island Quebec | | Saskatchewan | Territories |
| 2009 | - | √ | - | √ | - | - | - | - | √ | √ | √ |
| 2010 | - | √ | - | √ | - | - | - | - | √ | √ | √ |
| 2011 | - | √ | - | - | - | - | - | - | √ | - | √ |
| 2012 | - | √ | - | - | - | - | - | - | √ | - | √ |
| 2013 | - | - | - | - | - | √ | - | - | √ | - | - |
| 2014 | - | - | - | - | - | √ | - | - | √ | - | - |
| 2015 | √ | - | - | - | - | - | - | √ | - | - | √ |
| 2016 | √ | - | - | - | - | - | - | √ | - | - | √ |
| 2017 | √ | √ | - | - | √ | - | - | √ | - | - | - |
| 2018 | √ | √ | - | - | √ | - | - | √ | - | - | - |

| **Table S4** Average social support score across different subgroups populations, Canada, 2009-2018 | | | | | | | | | | | | |
| --- | --- | --- | --- | --- | --- | --- | --- | --- | --- | --- | --- | --- |
| Variable | 2009^#^ | 2010^#^ | 2011^*^ | 2012^*^ | 2013^*^ | 2014^*^ | 2015^*^ | 2016^*^ | 2017^*^ | | 2018^*^ | |
| National level | 65.8 | 65.8 | 31.5 | 35.7 | 35.9 | 35.9 | 35.3 | 35.0 | 34.9 | | 35.1 | |
| Gender |  |  |  |  |  |  |  |  |  | |  | |
| Men | 65.6 | 65.2 | 31.2 | 35.3 | 35.4 | 35.2 | 34.9 | 34.6 | 34.5 | | 34.9 | |
| Women | 66.1 | 66.3 | 31.8 | 36.1 | 36.3 | 36.5 | 35.8 | 35.5 | 35.4 | | 35.4 | |
| Age (years) |  |  |  |  |  |  |  |  |  | |  | |
| 12-19 | 68.2 | 68.7 | 31.6 | 36.1 | 36.3 | 36.5 | 35.7 | 35.3 | 35.4 | | 35.8 | |
| 20-29 | 67.6 | 67.0 | 31.9 | 36.5 | 36.9 | 37.0 | 36.0 | 35.7 | 35.7 | | 35.9 | |
| 30-39 | 66.4 | 66.2 | 31.8 | 36.3 | 37.0 | 36.7 | 35.3 | 35.7 | 35.4 | | 35.4 | |
| 40-49 | 65.0 | 65.5 | 31.3 | 35.7 | 36.2 | 36.1 | 35.3 | 34.7 | | 35.0 | | 34.8 |
| 50-59 | 64.6 | 64.5 | 31.5 | 35.5 | 35.4 | 35.4 | 35.3 | 34.7 | 34.3 | | 34.8 | |
| 60-69 | 65.3 | 64.2 | 31.3 | 35.2 | 34.9 | 35.0 | 35.0 | 34.3 | 34.5 | | 34.8 | |
| 70+ | 63.4 | 64.3 | 30.8 | 34.1 | 33.8 | 33.8 | 33.9 | 33.7 | 34.0 | | 34.2 | |
| Province of residence |  |  |  |  |  |  |  |  |  | |  | |
| AB | - | - | - | - | - | - | 35.3 | 35.0 | 34.8 | | 35.0 | |
| BC | 65.2 | 64.8 | 31.5 | 35.4 | - | - | - | - | 35.0 | | 35.1 | |
| MB | - | - | - | - | - | - | - | - | - | | - | |
| NB | 67.8 | 67.2 | - | - | - | - | - | - | - | | - | |
| NL | - | - | - | - | - | - | - | - | 35.5 | | 35.7 | |
| NS | - | - | - | - | 36.1 | 36.1 | - | - | - | | - | |
| ON | - | - | - | - | - | - | - | - | - | | - | |
| PEI | - | - | - | - | - | - | 36.9 | 35.6 | 35.7 | | 36.0 | |
| QC | 66.0 | 66.0 | 31.5 | 35.9 | 35.9 | 35.8 | - | - | - | | - | |
| SK | 66.0 | 67.3 | - | - | - | - | - | - | - | | - | |
| Territories | 68.2 | 62.6 | 30.9 | 35.4 | - | - | 36.0 | 35.8 | - | | - | |
| Residency area |  |  |  |  |  |  |  |  |  | |  | |
| Rural | 67.3 | 67.4 | 31.7 | 35.6 | 35.8 | 35.7 | 36.0 | 35.3 | 35.2 | | 35.1 | |
| Urban | 65.5 | 65.4 | 31.5 | 35.7 | 35.9 | 35.9 | 35.2 | 35.0 | 34.9 | | 35.1 | |
| Population centre size |  |  |  |  |  |  |  |  |  | |  | |
| Rural area | 67.5 | 67.3 | 31.6 | 35.6 | 35.8 | 35.7 | 35.9 | 35.3 | 35.0 | | 35.0 | |
| Urban core | 65.4 | 65.2 | 31.5 | 35.7 | 36.0 | 35.9 | 35.2 | 34.9 | 34.9 | | 35.1 | |
| Urban fringe | 67.0 | 67.4 | 31.5 | 36.3 | 34.6 | 36.0 | 36.3 | 35.0 | 35.3 | | 35.6 | |
| Urban O/S CMA | 66.9 | 66.1 | 31.6 | 35.9 | 35.5 | 35.4 | 35.0 | 35.2 | 35.0 | | 34.9 | |
| Secondary urban core | 63.6 | 67.9 | 31.2 | 36.7 | 35.1 | 36.1 | 35.6 | 36.0 | 35.3 | | 35.4 | |
| Mix of Urban/rural | 66.2 | 66.9 | 31.7 | 35.7 | 35.9 | 36.2 | 36.5 | 35.2 | 35.5 | | 35.3 | |

Note: # represents social support measurement is Social Support Availability for the year 2009/2010; * represents social support measurement is Social

Provisions Scale from 2010 onwards; AB: Alberta; BC: British Columbia; MB: Manitoba; NB: New Brunswick; NL: Newfoundland and

Labrador; NS: Nova Scotia; ON: Ontario; PEI: Prince Edward Island; QC: Quebec; SK: Saskatchewan.

| **Table S5** Proportions of a high level of social support, Canada, 2009-2018 | | | | | | | | | | |
| --- | --- | --- | --- | --- | --- | --- | --- | --- | --- | --- |
| Variable | 2009 | 2010 | 2011 | 2012 | 2013 | 2014 | 2015 | 2016 | 2017 | 2018 |
|  | P%  (95%CI) | P%  (95%CI) | P%  (95%CI) | P%  (95%CI) | P%  (95%CI) | P%  (95%CI) | P%  (95%CI) | P%  (95%CI) | P%  (95%CI) | P%  (95%CI) |
| National level | 30.2  (29.2, 31.3) | 32.9  (31.7, 34.0) | 65.4  (64.1, 66.6) | 80.6  (79.4, 81.6) | 80.3  (79.1, 81.4) | 80.4  (79.1, 81.6) | 69.9  (68.1, 71.6) | 76.9  (75.3, 78.4) | 77.1  (76.0, 78.1) | 78.3  (77.2, 79.3) |
| Gender |  |  |  |  |  |  |  |  |  |  |
| Men | 30.8  (29.3, 32.5) | 33.2  (31.5, 34.9) | 60.6  (58.7, 62.5) | 77.5  (75.6, 79.2) | 77.4  (75.5, 79.1) | 75.5  (73.5, 77.5) | 76.6  (74.0, 79.0) | 75.3  (73.0, 77.5) | 75.3  (73.8, 76.7) | 77.4  (75.8, 78.9) |
| Women | 29.7  (28.3, 31.1) | 32.6  (31.0, 34.2) | 70.0  (68.4, 71.5) | 83.6  (82.3, 84.8) | 83.0  (81.4, 84.5) | 85.0  (83.7, 86.3) | 81.8  (79.8, 83.7) | 78.5  (76.3, 80.5) | 78.9  (77.3, 80.3) | 79.2  (77.7, 80.6) |
| Age (years) |  |  |  |  |  |  |  |  |  |  |
| 12-19 | 30.8  (28.0, 33.8) | 32.3  (29.5, 35.2) | 69.8  (66.7, 72.7) | 86.3  (83.5, 88.7) | 88.3  (85.6, 90.6) | 88.3  (85.8, 90.4) | 84.9  (80.3, 88.6) | 80.3  (75.2, 84.5) | 84.3  (81.4, 86.8) | 85.5  (82.2, 88.3) |
| 20-29 | 31.8  (29.0, 34.7) | 33.3  (30.7, 36.1) | 72.6  (69.5, 75.6) | 86.5  (83.3, 89.2) | 87.4  (84.5, 89.9) | 89.7  (86.7, 92.0) | 84.6  (80.9, 87.6) | 82.0  (77.7, 85.7) | 83.5  (80.7, 86.0) | 85.3  (82.4, 87.8) |
| 30-39 | 30.2  (27.8, 32.8) | 33.7  (31.0, 36.6) | 69.9  (66.5, 73.1) | 84.8  (82.4, 87.0) | 86.6  (84.2, 88.7) | 84.9  (81.9, 87.5) | 77.6  (73.5, 81.2) | 81.3  (78.1, 84.0) | 80.6  (78.2, 82.8) | 80.0  (77.4, 82.4) |
| 40-49 | 28.5  (25.6, 31.6) | 33.3  (30.2, 36.5) | 61.6  (58.0, 65.2) | 78.7  (75.0, 81.9) | 81.4  (77.5, 84.7) | 81.0  (76.3, 84.9) | 79.7  (75.3, 83.5) | 74.9  (70.1, 79.1) | 74.6  (71.8, 77.3) | 74.7  (71.5, 77.7) |
| 50-59 | 28.8  (26.5, 31.3) | 31.6  (28.4, 34.9) | 64.9  (61.8, 67.9) | 78.3  (75.2, 81.1) | 75.3  (71.8, 78.5) | 76.5  (73.3, 79.5) | 76.2  (71.5, 80.4) | 73.7  (69.7, 77.4) | 72.4  (69.3, 75.4) | 75.1  (72.2, 77.8) |
| 60-69 | 32.5  (30.1, 35.0) | 33.1  (30.3, 36.0) | 61.1  (58.2, 63.9) | 76.2  (73.8, 78.5) | 73.4  (70.2, 76.3) | 73.5  (70.7, 76.1) | 78.6  (74.5, 82.3) | 69.4  (64.5, 73.8) | 72.9  (70.3, 75.4) | 75.2  (72.6, 77.7) |
| 70+ | 30.2  (27.6, 32.9) | 32.7  (29.9, 35.6) | 53.5  (50.4, 56.5) | 70.3  (67.5, 72.9) | 66.7  (63.6, 69.7) | 66.9  (63.7, 69.9) | 68.3  (62.7, 73.3) | 70.0  (65.7, 74.1) | 70.2  (67.3, 72.9) | 71.6  (68.8, 74.2) |
| Province of residence |  |  |  |  |  |  |  |  |  |  |
| AB | - | - | - | - | - | - | 78.8  (77.1, 80.4) | 76.7  (75.1, 78.3) | 77.2  (75.7, 78.7) | 77.7  (76.0, 79.3) |
| BC | 29.5  (27.7, 31.4) | 32.4  (30.3, 34.5) | 64.6  (62.5, 66.6) | 79.6  (77.5, 81.5) | - | - | - | - | 76.6  (74.9, 78.2) | 78.3  (76.7, 79.8) |
| MB | - | - | - | - | - | - | - | - | - | - |
| NB | 35.6  (32.7, 38.5) | 34.0  (31.2, 36.9) | - | - | - | - | - | - | - | - |
| NL | - | - | - | - | - | - | - | - | 79.9  (76.8, 82.6) | 81.3  (78.3, 83.9) |
| NS | - | - | - | - | 84.8  (82.5, 86.8) | 84.1  (81.8, 86.1) | - | - | - | - |
| ON | - | - | - | - | - | - | - | - | - | - |
| PEI | - | - | - | - | - | - | 89.1  (86.6, 91.2) | 81.0  (77.3, 84.3) | 80.4  (76.9, 83.5) | 83.0  (79.5, 86.1) |
| QC | 29.8  (28.3, 31.4) | 32.5  (30.9, 34.2) | 65.9  (64.4, 67.5) | 81.1  (79.7, 82.4) | 79.7  (78.4, 81.0) | 79.9  (78.6, 81.2) | - | - | - | - |
| SK | 32.0  (29.4, 34.7) | 37.1  (34.2, 40.1) | - | - | - | - | - | - | - | - |
| Territories | 44.5  (38.7, 50.3) | 33.7  (28.6, 39.2) | 57.3  (54.0, 60.5) | 81.7  (78.8, 84.3) | - | - | 81.5  (75.1, 86.6) | 81.5  (76.8, 85.4) | - | - |
| Residency area |  |  |  |  |  |  |  |  |  |  |
| Rural | 33.3  (31.2, 35.4) | 36.1  (33.8, 38.4) | 66.7  (64.4, 69.0) | 79.4  (76.9, 81.7) | 79.8  (77.9, 81.6) | 78.0  (75.8, 80.1) | 83.8  (80.9, 86.3) | 79.1  (76.2, 81.8) | 78.3  (76.3, 80.2) | 78.0  (75.7, 80.1) |
| Urban | 29.5  (28.3, 30.7) | 32.1  (30.8, 33.4) | 65.1  (63.7, 66.5) | 80.8  (79.5, 82.0) | 80.4  (78.9, 81.8) | 81.1  (79.7, 82.5) | 78.3  (76.5, 80.1) | 76.5  (74.7, 78.2) | 76.9  (75.7, 78.0) | 78.3  (77.1, 79.5) |
| Population centre size |  |  |  |  |  |  |  |  |  |  |
| Rural area | 34.1  (31.8, 36.6) | 35.5  (32.9, 38.1) | 66.4  (63.9, 68.8) | 79.3  (76.6, 81.7) | 79.7  (77.7, 81.5) | 78.0  (75.7, 80.1) | 82.6  (79.2, 85.5) | 79.7  (76.5, 82.6) | 76.7  (74.1, 79.0) | 77.5  (75.2, 79.7) |
| Urban core | 29.3  (28.0, 30.6) | 31.7  (30.3, 33.2) | 64.8  (63.3, 66.4) | 80.6  (79.2, 81.9) | 80.8  (79.2, 82.3) | 81.2  (79.5, 82.7) | 78.5  (76.4, 80.4) | 76.2  (74.1, 78.1) | 76.5  (75.2, 77.8) | 78.1  (76.8, 79.4) |
| Urban fringe | 33.4  (26.7, 40.8) | 37.3  (29.7, 45.5) | 65.1  (55.6, 73.5) | 85.2  (78.2, 90.3) | 73.1  (64.4, 80.4) | 81.2  (75.5, 85.9) | 81.7  (70.3, 89.4) | 73.6  (61.4, 83.0) | 79.6  (72.5, 85.2) | 83.0  (76.9, 87.8) |
| Urban O/S CMA | 30.8  (26.9, 34.9) | 36.8  (32.2, 41.6) | 67.5  (63.1, 71.6) | 81.9  (78.4, 85.0) | 79.2  (75.2, 82.7) | 79.1  (74.8, 82.8) | 75.7  (70.8, 80.0) | 78.2  (74.0, 82.0) | 78.4  (75.3, 81.1) | 78.1  (74.7, 81.2) |
| Secondary urban core | 28.7  (20.4, 38.6) | 33.2  (24.6, 43.3) | 62.5  (52.0, 72.0) | 86.1  (78.8, 91.1) | 80.2  (71.7, 86.6) | 81.3  (72.7, 87.6) | 77.9  (67.3, 85.8) | 81.4  (74.5, 86.7) | 80.8  (75.3, 85.3) | 79.7  (73.0, 85.1) |
| Mix of Urban/rural | 30.2  (27.0, 33.5) | 34.3  (30.6, 38.3) | 68.9  (64.8, 72.8) | 79.5  (73.8, 84.2) | 79.4  (69.9, 86.6) | 89.3  (80.9, 94.3) | 87.6  (81.8, 91.8) | 77.5  (70.6, 83.1) | 81.2  (77.7, 84.2) | 78.8  (73.8, 83.1) |

Note: P: point estimate; 95%CI: 95% confidence interval; AB: Alberta; BC: British Columbia; MB: Manitoba; NB: New Brunswick; NL: Newfoundland and Labrador; NS: Nova Scotia; ON: Ontario; PEI: Prince Edward Island; QC: Quebec; SK: Saskatchewan; - no data available.

**Fig. S1** Average life satisfaction score, Canada, 2009-2018

**Fig. S2** Average life satisfaction score by provinces and territories, Canada, 2009-2018

**Fig. S3** Average life satisfaction score by different age groups, Canada, 2009-2018

**Fig. S4** Average life satisfaction score by different population size of residential areas, Canada, 2009-2018

**Fig. S5** Age-and gender- standardized point estimate of life satisfaction scored nine and ten and its 95% confidence interval, Canada, 2009-2018

**Fig. S6** Age-and gender-standardized point estimate of life satisfaction scored less than six and its 95% confidence interval, Canada, 2009-2018


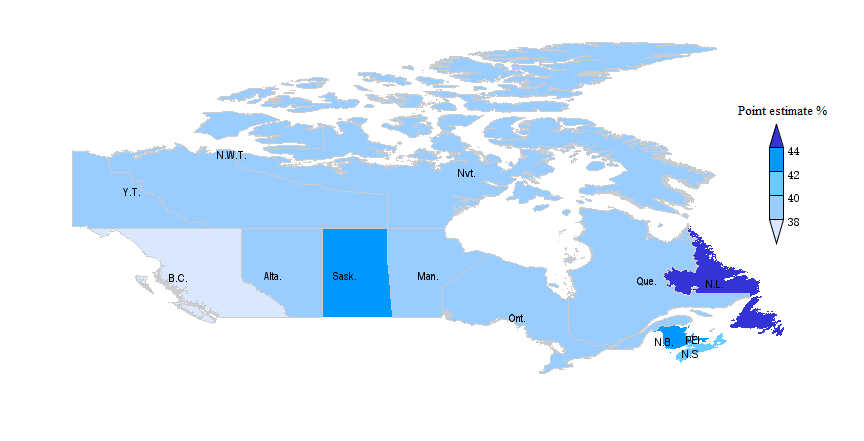


**Fig. S7** The point estimates of life satisfaction scored as nine or ten by provinces and territories, Canada, 2009-2018

Note: Alta.: Alberta; B.C.: British Columbia; Man.: Manitoba; N.B.: New Brunswick; N.L.: Newfoundland and Labrador; N.S.: Nova Scotia; Ont.: Ontario; P.E.I.: Prince Edward Island; Que: Quebec; Sask.: Saskatchewan; Y.T., N.W.T., and NvT.: Territories.


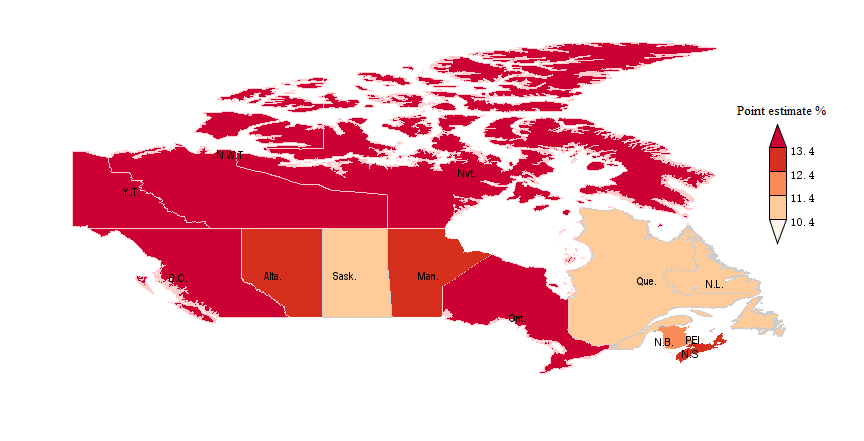


**Fig. S8** The point estimates of life satisfaction scored as six or less by provinces and territories, Canada, 2009-2018

Note: Alta.: Alberta; B.C.: British Columbia; Man.: Manitoba; N.B.: New Brunswick; N.L.: Newfoundland and Labrador; N.S.: Nova Scotia; Ont.: Ontario; P.E.I.: Prince Edward Island; Que: Quebec; Sask.: Saskatchewan; Y.T., N.W.T., and NvT.: Territories.
